# The impact of the COVID-19 pandemic on illicit drug supply, drug-related behaviour of people who use drugs and provision of drug related services in Georgia: results of a mixed methods prospective cohort study

**DOI:** 10.1186/s12954-022-00601-z

**Published:** 2022-03-09

**Authors:** David Otiashvili, Tamar Mgebrishvili, Ada Beselia, Irina Vardanashvili, Kostyantyn Dumchev, Tetiana Kiriazova, Irma Kirtadze

**Affiliations:** 1 Addiction Research Center Alternative Georgia, 14A Nutsubidze Street, Office 2, 0177 Tbilisi, Georgia; 2 School of Natural Sciences and Medicine, Ilia State University, 3/5 Kakutsa Cholokashvili Ave., 0162 Tbilisi, Georgia; 3 School of Arts and Sciences, Ilia State University, 3/5 Kakutsa Cholokashvili Ave., 0162 Tbilisi, Georgia; 4 Ukrainian Institute On Public Health Policy, 5 Biloruska Str. Office 20, 27, Kyiv, 04050 Ukraine

**Keywords:** COVID-19, Illicit drug supply, Drug-use behaviour, Georgia

## Abstract

**Background:**

This study examines the effects of COVID-19 related restrictions on the supply of illicit drugs, drug-use behaviour among people who use drugs (PWUD) regularly (at least weekly), and drug-related service provision in Tbilisi, Georgia.

**Methodology:**

In this mixed methods study, a cohort of 50 Georgian PWUD recruited through a snow-ball sampling participated in a bi-weekly online survey in April–September, 2020. They also took part in the qualitative telephone interviews at 12- and 24-week follow-up time points. In addition, four key informants (field experts) were interviewed monthly to assess their perceptions of changes in the illicit drug market and drug service delivery.

**Results:**

Mean age in the sample was 36 (range 18–60); 39 (78%) were males. Perceived availability of drugs was reduced during the lockdown, and many PWUD switched to alternative substances when preferred drugs were not available. On average, participants used significantly fewer substances over the course of the study, from 3.5 substances in the preceding 14 days to 2.1 (aOR 0.92; 95% CI 0.90–0.94). Consumption of cannabis products declined significantly (aOR 0.89; 95% CI 0.84–0.95), likewise alcohol (aOR 0.94; 95% CI 0.88–1.0), diverted medicinal methadone (aOR 0.85; 95% CI 0.8–0.9) and diverted medicinal buprenorphine (aOR 0.91; 95% CI 0.84–0.99). PWUD cited fewer contacts with drug dealers, the lack of transportation, and the lack of conventional recreational environment as the main reasons for these changes. When access to sterile injection equipment was limited, PWUD exercised risk-containing injection behaviours, such as buying drugs in pre-filled syringes (aOR 0.88; 95% CI 0.80–0.96). Harm reduction and treatment programs managed to adopt flexible strategies to recover services that were affected during the initial stage of the pandemic.

**Conclusions:**

COVID-19-related restrictive measures mediated specific changes in supply models and drug-use behaviours. While adjusting to the new environment, many PWUD would engage in activities that put them under increased risk of overdose and blood-borne infections. Harm reduction and treatment services need to develop and implement protocols for ensuring uninterrupted service delivery during lockdowns, in anticipation of the similar epidemics or other emergency situations.

**Supplementary Information:**

The online version contains supplementary material available at 10.1186/s12954-022-00601-z.

## Introduction

The COVID-19 pandemic led to introduction of infection containment measures on both global and national levels. Restrictions on travel, physical distancing, business closures, and other measures affected many aspects of people’s lives. Growing evidence indicates that these measures have had a disruptive impact on illicit drug markets, affecting the availability and accessibility of drugs [1, 2]. People who use drugs (PWUD) may respond to such changes in drug markets and extraordinary social and economic situations by switching to new drugs, or modifying their drug-use patterns. These changes in the drug use can lead to increased health risks. For example, PWUD, while adhering to the rules of quarantine and restrictions on movement, can try purchasing drugs in larger quantities, and then consume them in larger quantities, or they may switch to more potent substances [3]. This can increase the risk of overdose, specifically given the higher probability of using drugs alone [4–6]. Those who consumed drugs largely in nightlife settings might reduce their use of specific substances (such as alcohol, MDMA and cocaine) and switch to alternative substances [7, 8]. With regard to drug supply during the pandemic, contactless drug dealing and online drug markets seem to play a more prominent role at the retail level [9, 10]. Regional reports, specifically from Eastern Europe, indicate that drug market disruptions may have resulted in increased small-scale clandestine production—and use—of amphetamine-type stimulants [1]. Requirements for physical distancing and other COVID-19 prevention measures (such as reduced working hours, limited staffing, lack of transportation) may have resulted in additional challenges for PWUD to access health care, including substance-use-related treatment and harm reduction services [11]. In many countries, provision of harm reduction services was temporarily closed or reduced during the COVID-19 lockdowns [12–15].

### Drug situation in Georgia prior to COVID-19

In Georgia, the majority of drug-related data concern injection drug use. The research interest (often driven by the priorities of international donors) and public health focus has been directed towards the most risky pattern of drug consumption. With an estimated 52,000 people who inject drugs (PWID), the prevalence of injection drug use in Georgia (2.2% of adult population) ranks among the highest in the world [16, 17]. In the most recent decade, the problematic/high risk drug use patterns can be characterized as polydrug injection use of opioid products, such as heroin and buprenorphine, and stimulants with a substantial share (however, declining in recent years) of home-made preparations [18]. In response, provision of treatment for substance use disorders and HIV prevention interventions, including low threshold harm reduction services, has expanded in the past decade. Across the country, harm reduction services for PWID are available through 18 fixed harm reduction sites and eight mobile van-based laboratories. These facilities carry out HIV and Hepatitis C (HCV) and B (HBV) testing, and also provide needle and syringe exchange services [18]. In 2019, such services (testing and counselling, needle and syringe distribution, and other auxiliary services) were provided to about 35,000 PWID. In 2019, out of an estimated 20,000 opioid-dependent individuals in Georgia, more than 14,000 received opioid agonist treatment (OAT), either with methadone or buprenorphine [18]. Importantly, the data on non-injection drug use in Georgia are limited. Available research indicates growing use of new psychoactive substances (NPS), which are increasingly available in the illicit market [19], and the increasing use of psychoactive substances in nightlife settings [20, 21].

Traditional distribution schemes for conventional drugs in Georgia (face-to-face contact with a dealer) have been influenced by small-scale “freelance” dealing and massive kitchen-based self-production of drugs [22]. However, recent studies suggest the diffusion of alternative distribution models. Mobile phone applications (e.g., *Telegram*) and online markets (e.g., *Matanga*) are now used for the drug procurement [23]; about a quarter of respondents in a recent study report purchasing their drugs via the Internet [19]. The virtual dealers employ a common modus operandi—upon the receipt of the payment they provide the consumers with coordinates and a photo of the place where their purchase (the drug) has been hidden in advance (dead drop). Finally, social supply plays a prominent role in the Georgian drug scene. Individuals who use cannabis, those using NPS, and festival and nightclub attendees report that in a majority of cases drugs were obtained from friends [19, 24, 25].

The first case of COVID-19 in the country was documented on March 5, 2020. During March–May 2020, the government instituted a number of measures intended to prevent the transmission of SARS-COV2. Restrictive measures included some form of lockdown, border closings, restriction of movement, closure of businesses and educational institutions, and curfew. These measures initially helped to keep the incidence of new COVID-19 infections relatively low. The restrictions were gradually removed starting from June 2020, but were partially reintroduced in November 2020 in response to a surge in new infections.

To our knowledge, the modern research aiming to understand the impact of COVID-19 pandemic on drug markets, patterns of drug consumption and drug-related service provision has mostly relied on cross-sectional data gathered through one-time online surveys among samples from general population or specific groups of PWUD. These findings therefore are limited to the specific time points of the lockdown and do not provide understanding of medium-term trajectories of drug consumption. In most cases, findings are further subject to limitations and biases associated with online recruitment and data collection [26, 27], but also with retrospective examination of behaviours [28]. The current study aimed to examine changes in drug taking behaviours, drug supply and drug-related service delivery over the first six months of the pandemic through collecting prospective longitudinal data from the cohorts of PWUD who consumed drugs regularly in Georgia and Ukraine. The quantitative data were combined with qualitative methods to enrich our understanding of the context and mechanisms of the observed changes and to possibly identify factors that may have contributed to those changes. This article reports findings from the Georgian study cohort.

## Methods

### Study procedures

We utilized a sequential explanatory design for this mixed methods study in which the qualitative data were used to explore quantitative findings [29, 30]; quantitative data were collected first and guided qualitative data collection. This approach ensures complementarity of the results from one method with the results from the other, where qualitative results provide contextual understanding of the processes underlying the quantitative outcomes and explain quantitative observations [31, 32]. Quantitative and qualitative data were analysed separately, and the integration occurred during the results presentation and interpretation, so that the findings from the two methods interweave within specific subsections each of which focuses on one aspect of the research question [33, 34].

### PWUD participants

The research team used a purposive snow-ball sampling with eight relatively heterogenous seeds—individuals who use drugs through injection and non-injection routes and individuals who use drugs in nightlife settings, including representatives of the LGBTQ community—to recruit PWUD in Tbilisi. Seeds were identified through the outreach workers of low threshold HIV prevention programs. The seeds recruited eligible participants using a chain referral sampling (snow-ball) approach. Communication with seeds and study participants, the entire process of recruitment and data collection was done through mobile phones and/or online platform. The research team has never met face-to-face with initial seeds or the rest of the PWUD sample. We determined eligibility through the phone interviews and relied on the extensive experience of the research team in this process. Apart from the comprehensive screening instrument, researchers used probes (e.g. dosing, frequency of use, consumption methods and routes, methods for drug preparation), cross-check questions, specific terminology (slang, street terms) to be assured that the individual was eligible for the study. The sample size was determined based on the available funding to support the research staff and provide incentives to the study participants. Regular drug use was defined as at least weekly use of a psychoactive substance (except cannabis or alcohol) in the prior six months**.** Other eligibility criteria included being (1) 18 years or older; (2) fluent in Georgian; (3) having reliable Internet access; (4) being willing to provide their cell phone number for communication and research data collection (interviews). We excluded those PWUD whose sole use of illicit substance was cannabis. Cannabis use in combination with other illicit drugs, use of licit psychoactive substances (prescription psychotropic medications and antihistamines with psychoactive effect), and use of alcohol was of interest for the study, and relevant data were collected. Therefore, whenever cannabis use is reported in the results, it is in all instances a combination use with other illicit psychoactive substance(s). To collect quantitative bi-weekly data, we used a free online platform *Kobotoolbox*. Participants had a 3-day window period to log in and complete the survey. In the morning on every data collection time point, each participant received SMS-reminders with the survey link, session number and participant ID. The study team used a locally developed structured 39-item (Q16-39 for the follow-up) questionnaire that covered participant demographics, types and frequency of drug use, supply methods, drug prices, perceived changes in quality and availability of drugs, and participants’ utilization of drug-related health services. The questionnaire incorporated measures from standardized instruments: Timeline Follow Back (TLFB) [35, 36], Addiction Severity Index (ASI) [37], and a risk behaviour questionnaire used for the Integrated Bio-Behavioural Surveillance Surveys (IBBS) in Ukraine [38]. The questionnaire is available in the Additional file 1. Prior to launch, the survey tool was test-piloted with the PWUD who had similar characteristics as the potential study participants. Demographic information included age, gender, education, employment status, and source of income. Drug use was measured through asking which drugs were used “ever” in the life time, in the past 12 months, and in the past 14 days; these past 14-day questions were repeated during each subsequent follow-up interview. Other outcomes of interest included source(s) of drugs, perceived changes in their price and quality, frequency of use, history of overdose, access and use of treatment and harm-reduction services. Details of outcome variables, measures and assessment time points are presented in Additional file 1: Table S1. To collect the qualitative data, two research assistants conducted telephone interviews with all PWUD cohort participants at 12- and 24-week time points. The interviewer covered topics related to the perceived changes in drug supply and drug-use behaviours, and factors influencing them. With the consent of respondents, the interviews were digitally recorded. Timeline of the data collection events is presented in Additional file 1: Fig. S1.

### Key informant participants

Apart from the PWID cohort, four key informants were recruited from the pool of individuals working in the field of psychoactive substance use in Tbilisi. Final composition of the key informants’ group was: head of a harm reduction site, head of an OAT site, head of a private detoxification clinic, and head of a PWUD community based organization. The key informants were interviewed by the first author at the end of each study month (six interviews with each respondent) via phone and were asked to provide information about changes in drug market, drug-user behaviour and service delivery during the preceding month. The qualitative interview guide covered the following domains: drug markets (availability, supply channels, quality, price); behaviour of PWUD with regard to drug procurement and consumption; risk-containing behaviours; availability and use of drug-related health services. Interviews typically lasted 15–30 min and were recorded with the permission of the respondents. Key informants were not the part of the PWUD cohort, so no data collected from key informants were used in quantitative analysis.

### Data analysis

The sociodemographic and behavioural profile of PWUD study participants was described using frequencies and proportions (for categorical variables), and mean values for continuous variables, as appropriate. The original study protocol foresaw testing of hypothetical inflection points—corresponding to the major disruption or change in social patterns caused by the pandemic—for the key variables of interest, using spline regression models or similar methods. However, an interim analysis revealed that the use of different substances and other variables changed inconsistently over time, and a joint hypothesis could not be defined. As an alternative, we chose a hypothesis of linear trend, to assess whether substance use, behaviours and services increased or decreased significantly during the study period. To account for within-subject autocorrelation across time points, we used mixed effects generalized linear models. The assessment number, ranging from 1 to 13, was used as a continuous variable representing time. Adjusted Odds Ratios (aORs) for the assessment number represent an incremental increase or decrease in the estimated likelihood of achieving the outcome in the subsequent assessment; significant aORs indicate a decrease or increase in the given variable over the study period. The models were adjusted for the duration of drug use, sex, and baseline lifetime exposure to OAT. The analysis was done using R software version 4.0.3 [39].

For the qualitative component of the study, two research assistants (the same who conducted qualitative telephone interviews) transcribed recordings verbatim. Data were analysed using NVivo v.11 software for qualitative analysis [40]. We relied on a framework analysis approach for this study [41]. The framework method is an effective tool to support thematic (qualitative content) analysis and is most suitable for the analysis of the interview data, where researchers expect to generate themes by making comparisons within and between cases. Importantly, framework method facilitates management of large data sets through its matrix form which provides an intuitively structured overview of the summarized data. Following the reading and rereading of the textual data, the list of key themes was developed. A set of codes that were organized into categories was agreed and applied to all transcripts. However, if new ideas and new ways of categorizing were identified in the text, the list of hierarchical codes was amended. This approach helped to identify commonalities and differences in data and to draw descriptive and explanatory conclusions clustered around themes.

### Attrition

There were 13 sessions for the online quantitative survey with PWUD, conducted every other week starting on April 7 and ending on 24 September 2020. One participant dropped out after the sixth session. Overall, of the 650 planned sessions, 40 sessions (6.15%) were missed. There were 40 individual qualitative interviews conducted with PWUD participants at the 12-week follow-up time point, and 34 interviews conducted at the 24-week follow-up. No interviews were missed with the key informants—in total, there were 24 individual telephone interviews conducted with the drug field experts (four interviews each month).

### Ethical aspects of the study

This study was approved by the Bio-Ethics Committee of the School of Arts and Sciences at Ilia State University in Tbilisi, Georgia. Digital informed consent was obtained from all respondents. No identifiable personal information was collected from the PWUD cohort participants. PWUD respondents were compensated for their time and the cost of Internet use/access. Datasets for the current work are publicly available through Mendeley Data [42].

## Results

Mean age of PWUD participants was 36 (range 18–60), of whom 39 (78%) were males, 20 (40%) had a university degree, and slightly more than a half were employed at baseline. Full- or part-time employment as the main source of income was reported by 27 participants (54%) at baseline. Seventeen participants (34%) had a history of at least one treatment episode for substance-use-related disorders. Socio-demographic characteristics of PWUD participants are given in Table 1.

**Table 1:** Socio-demographic characteristics of participants at baseline (*N* = 50)

Variable	N *(%)*
*Age*	
15–19	1 (2)
20–24	6 (12)
25–29	8 (16)
30–34	7 (14)
35–39	11 (22)
40–44	7 (14)
45–49	6 (12)
50–54	2 (4)
55–59	1 (2)
60–64	1 (2)
Mean (SD)* (min, max)	36 (9.88); SE (± 1.4); (18, 60)
*Sex*	
Male	39 (78)
Female	10 (20)
Nonbinary	1 (2)
*Education*	
Incomplete high school	5 (10)
Completed high school	8 (16)
Completed high school-vocational	4 (8)
Incomplete university	13 (26)
Completed university	20 (40)
*Employment status*	
Employed	27 (54)
Student and employed	2 (4)
Retired/social benefit	1 (2)
Unemployed	20 (40)

*Standard deviation

Five participants in the study cohort were on OAT at baseline, and stayed in treatment until the end of the data collection. Six participants initiated OAT during the study. Over the course of the study, 27 participants reported having being tested for COVID-19 and none was positive.

### Drug use

**Fig. 1:**
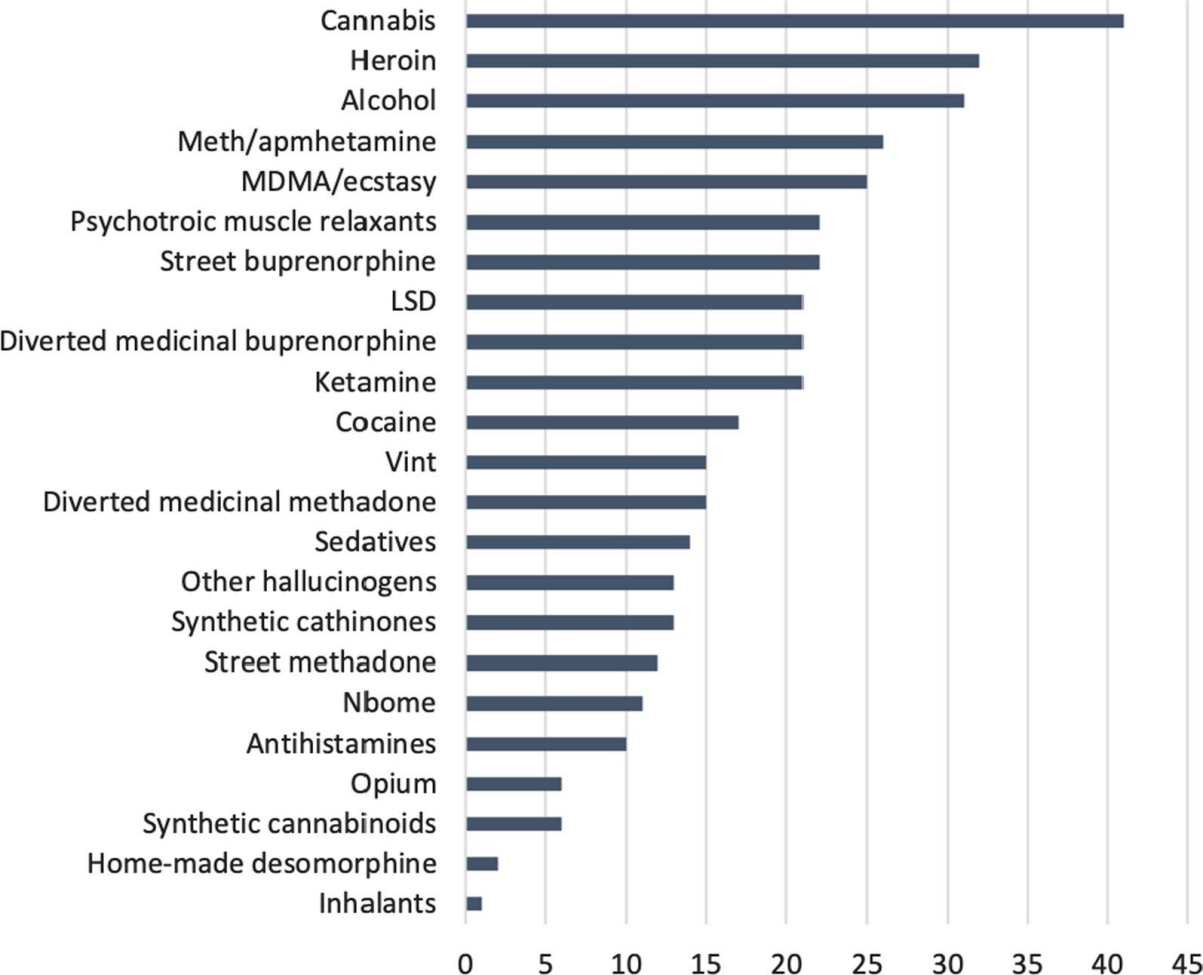
Proportion of respondents (%) reporting at baseline the use of particular drugs in the past 12 months (*N* = 50). *Note*: the sum exceeds 100% due to the use of multiple substances by individual participants; Vint refers to a home-made long-acting stimulant prepared through the reduction of pseudoephedrine

At baseline, cannabis products were the most frequently used substances in the preceding 12 months. These were followed by heroin and alcohol. Use of amphetamine/methamphetamine and MDMA/ecstasy was also relatively high (see Fig. 1).

**Fig. 2:**
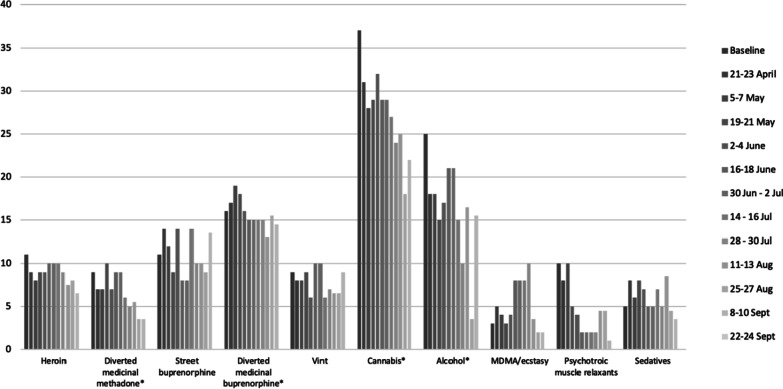
Number of respondents reporting the use of particular drugs in the past 14 days (10 most prevalent drugs) (*N* = 50), April–September, 2020. *Note*: the sum exceeds *N* = 50 due to the use of multiple substances by individual participants; *-reduction in the use was statistically significant; exact results of the tests are shown in Table 2

There was a number of observable trends in relation to the past 14-day consumption of various substances during the period studied, as shown in Fig. 2. The frequency of use of cannabis products gradually decreased over the course of the study, from 74% (*n* = 37) at baseline to 44% (*n* = 22) by the end of September. Alcohol was the second most frequently consumed substance at baseline (50%, *n* = 25), and its consumption declined in April–May, 2020 (the strictest lockdown period), but subsequently returned nearly to baseline levels in June when restrictions were largely removed, and again declined by the end of September 2020 (31%).

Buprenorphine diverted from OAT programs was the third most prevalent substance used at baseline. Analysis via mixed effect models indicates a statistically significant descending trend in consumption of cannabis products, alcohol, amphetamines, diverted medicinal methadone, diverted medicinal buprenorphine, Ketamine, LSD and myorelaxants (see Table 2). The number of different substances used by the participants also declined significantly over the course of the study, from on average 3.5 substances in the preceding 14 days to 2.1 (aOR 0.92; 95% CI 0.90–0.94).

**Table 2:** Statistically significant results of mixed effect models^a^ for testing an association between specific outcomes of interest and time (assessment number)

Variable	aOR^b^	*p* value	95% CI
Having full- or part-time job	1.21	0.000	1.12–1.30
Receiving financial support from others	0.87	0.000	0.81–0.93
Used medicinal methadone	0.85	0.000	0.78–0.93
Used medicinal buprenorphine	0.91	0.022	0.84–0.99
Used amphetamine	0.78	0.001	0.68–0.90
Used cannabis	0.89	0.001	0.84–0.95
Used alcohol	0.94	0.049	0.88–1.00
Used ketamine	0.83	0.001	0.74–0.93
Used LSD	0.87	0.013	0.79–0.97
Used myorelaxants	0.77	0.000	0.69–0.87
Used medicinal methadone by injection	0.89	0.023	0.80–0.98
Used medicinal buprenorphine by injection	0.87	0.001	0.80–0.95
Use of fewer different substances	0.92	0.001	0.90–0.94
Price of drugs become more expensive	0.88	0.000	0.82–0.94
Access to drugs become harder	0.89	0.000	0.83–0.95
Access to harm reduction services became harder	0.66	0.000	0.57–0.78
Received drug in prefilled syringe	0.88	0.006	0.80–0.96

^a^The models for each outcome of interest were adjusted for the effects of sex, duration of drug use, and lifetime exposure to opioid agonist therapy and included participant ID as a random effect

^b^The adjusted odds ratios show by how much the odds of the outcome will increase (aOR > 1) or decrease (aOR < 1) with each consecutive assessment

Results describing frequency of drug use were mixed, with some respondents reporting higher frequency of consumption at some points, and others reporting lower frequency of use. Overall, we did not observe any statistically significant trends in the frequency of drug consumption. Similarly, the results with regard to amounts of drugs consumed were mixed.

Qualitative interviews provided useful insight into the changes in drug use behaviour of study participants. The reduced availability of preferred substances in some instances caused users to look for alternative substances. In some cases, these were substances that were familiar to individuals and had been used at some point earlier in their drug history. In other cases, individuals switched to the substances that they had never tried before. Among other factors shaping these trends, respondents named stress and anxiety surrounding the first weeks of COVID-19 cases in the country and the “social panic” that accompanied it:

*...due to lockdown, people started using such substances that they almost never used in other times. … I myself did not try vint for [a] couple years and used it now few times, I would never do that if not for those lockdown.*

In addition, many individuals consumed only substances that were readily available to them:

*I use now what is available. For example, a lot of cannabis and Ketamine. I have never used Ketamine so often. I now tried vint as well. I would not use it if other drugs were available.*

Some respondents referred to “*plenty of free time and boredom*”, and stressful context of the epidemic as contributing factors leading to increased frequency of drug use:

*I had lot of free time, there was nothing to do, no hanging out with people, and lot of depression around, so you need more drugs to deal with depression… I can say that I used vint and psychedelics more often at that time, and smoked pot really too much.*

On the other hand, those who reported reduced frequency of drug use referred to difficulties in obtaining drugs, high prices and low quality as their main reasons. For some users, the principal reason for less frequent use was the closure of nightclubs and other spaces where they frequently consumed drugs:

*I usually used drugs in clubs, sometimes at home. I was buying for overnight club use. Now clubs are closed and I even do not buy drugs and only use when someone offers.*

As *o*ne key informant noted, “*it makes no sense to use ecstasy at home, so you better use LSD or ketamine*”.

### Price, quality and availability of drugs

In the quantitative online survey, the majority of participants reported relatively stable quality (based on their perceptions) and price for most drugs over the course of the study. However, in April–May, 40% of participants believed that the price of their preferred drug increased. Over the course of the study, the proportion of participants reporting their perception of price increases gradually declined. In other words, with each consecutive assessment, more respondents indicated that the price of their main drug did not increase, and this trend was statistically significant (aOR 0.88; *p* = 0.000; 95% CI 0.82–0.94)—see Table 2. When looking into the data on exact prices paid by participants for various drugs, the picture is heterogenous. Throughout April–September, price paid for a single dose of a specific drug increased to different degree for heroin, diverted medicinal methadone, and psychotropic sedatives. Prices trended downward for street methadone, street buprenorphine, cocaine, cannabis products, ketamine and MDMA/ecstasy. However, these trends were not statistically significant.

The quantitative data suggest that the perceived availability and access to the main drug of choice were strongly affected, particularly during the strictest lockdown period in April–May, 2020. In Session 2 (end of April, 2020), the majority of respondents (55%) believed that it was harder to obtain drugs compared to previous periods. However, towards the end of the study, an increasing number of respondents reported that obtaining their main drug of choice was not getting more difficult. This trend was statistically significant (aOR 0.89; *p* = 0.000; 95%CI 0.83–0.95).

In the qualitative interviews, the opinions about changes in price and quality of drugs were ambiguous, with some respondents believing that the price for most substances increased steeply during the lockdown:

*Absolutely everything [is more expensive now], except for opiates. You would buy MDMA for 250 GEL, but if you can get it now, it costs 400-500 GEL. Ecstasy is now 120-150 GEL and you are lucky if it’s normal, but we used to buy it for 60-80 GEL.*

With regard to access to drugs, for many respondents, the reduction in the availability of drugs was particularly evident for stimulants and cannabis products. Others suggested that the most affected (in terms of availability) substance was heroin, which was largely procured via person-to-person contacts with dealers. Further, respondents noted that there were fewer sellers and a reduced variety of drugs offered through online markets. The qualitative interviews further indicate that participants observed an increase in the availability of methadone and buprenorphine diverted from OAT programs. As stated by the respondents, these were both sold by OAT clients and supplied through friendship networks for free:

*Suboxone and methadone from programs were easy to get, they [*patients*] received medication to take home and they easily sold it.*

The participants also suggested that some sellers made adjustments with regard to places where dead drops were hidden. In many cases locations for dead drops moved to Tbilisi suburbs, quiet places where there was a less police movement and surveillance. In some cases, when drugs were bought from online markets, the dead drops were not in places indicated by online sellers, or a customer could not access the location. Often, it was difficult to move in the city due to a lack of transportation options, restrictions that were introduced by the government. The police presence on the streets also increased, hence going to pick up dead drops and moving with drugs through the city was perceived as more challenging and risky.

Since established contacts with dealers were affected, some individuals started acting as middleman dealers (called  (phekhi)*—“*foot” in Georgian). For example, users who were able to pay for drugs and who wanted to avoid excessive in-person contacts, employed other users who would take the risk associated with drug delivery in exchange of a personal dose. According to one respondent, “*…my dealer disappeared, so I found someone who knows another dealer, and this middleman brings me drugs for a cut”.*

Finally, participants believed that in July–September 2020, when COVID-19-related restrictions were eased, the drug market started returning to “*business as usual*”. Heroin was cited as an example—with the partial removal of COVID-19 related restrictions, it became available again through traditional person-to-person transactions: *“…trade goes on as normal, hands to hands in Phonichala [*district of Tbilisi*]”.*

### Means of obtaining drugs

Results of the quantitative survey demonstrate that there were remarkable variations in the ways specific drugs were acquired by study participants. At various data collection time points, between 50 and 100% (*N* = 25 to 50) of respondents who used diverted medicinal methadone and buprenorphine reported buying them from friends or receiving them for free from friends. Free of charge supply to social network members was particularly typical for cannabis products—at each data collection session, at least 40% (maximum 70%) of cannabis users reported receiving it for free from friends. Throughout the study period, cocaine was purchased largely via mobile applications which were also frequently used to procure Ketamine, MDMA, NBOMe and meth/amphetamines. Antihistamines (used in combination with opioids to enhance and prolong their effect), psychotropic muscle relaxants, and sedatives were mostly obtained from pharmacies. We did not find any statistically significant changes in how any specific drug was acquired over the study period.

In qualitative interviews, many respondents stated that they were unable to keep stable contact with the dealers, so they had to look for new sources repeatedly:

*I can say that we look for new channels almost every day. Trying this, trying that. Nowadays I don’t know a stable dealer who stays active and has a stable supply.*

In doing so, participants explored novel schemes for purchasing drugs during the lockdown period. For example, it was possible to receive drugs delivered to your home:

*If previously I was going to dealer’s place, now he brings the drug to my place*.

### Risk-taking behaviour

**Fig. 3:**
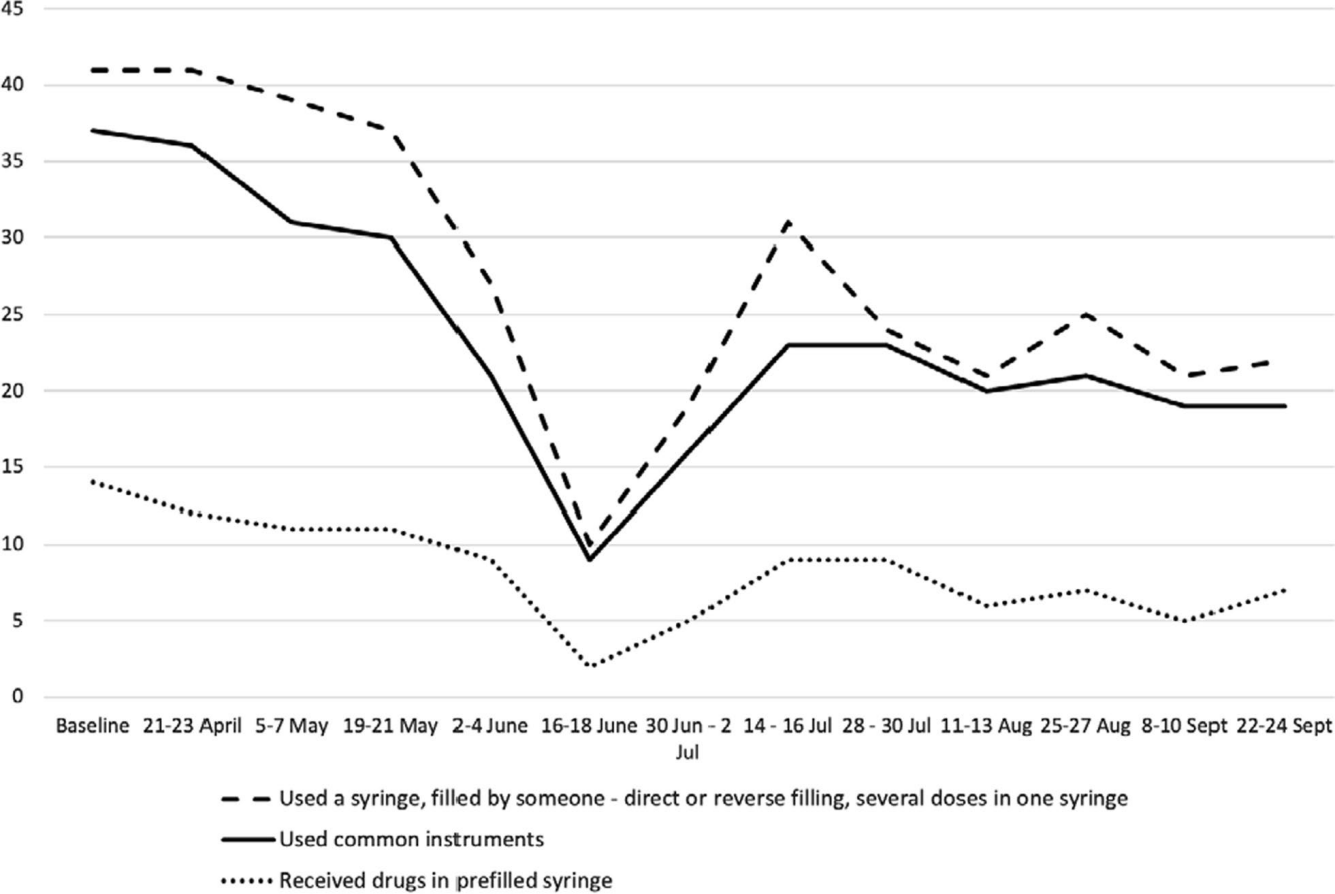
Number of respondents reporting a specific injection risk behaviour in the past 14 days (*N* = 50), April–September, 2020

About 80% of the participants reported injection drug use over the study period. The quantitative survey included several questions covering potentially high-risk injection behaviours. The most prevalent way to obtain sterile equipment throughout the study was a purchase from a pharmacy—at various data collection time points, 35% to 62% of respondents reported buying syringes in pharmacies***.*** During the first weeks of the lockdown, up to 30% of participants reported receiving used needles and syringes from others (typically a friend or a partner, see Fig. 3 for details).

During the same period of the strictest lockdown (April–May, 2020), more than a quarter of participants reported obtaining drugs in pre-filled syringes. Another risk behaviour reported by participants was a direct and reverse filling (front and back loading) of syringes (up to 20%). However, the most prevalent risky practice was related to sharing common instruments for preparation and injection of drugs—see Fig. 3. During the strictest lockdown, almost half of the sample did share equipment with others at least once in the preceding 14 days. Sharing practices ceased as soon as lockdown measures were eased, and access to sterile equipment was restored. In a mixed effect model analysis, the odds of receiving drugs in a pre-filled syringe decreased with each consecutive assessment (aOR 0.88; *p* = 0.006, 95% CI 0.80–0.96)—see Table 2. Over the same period, increased odds of always having a new syringe for injection was marginally significant (aOR 1.1; *p* = 0.05, 95% CI 1.0–1.2) (data not shown).

### Availability and demand for services

Around two thirds of participants reported using some harm reduction services while participating in the study. Participants acknowledged that access to programs was particularly affected during the first two months of the study (April–May, 2020—the strictest lockdown period), and then gradually recovered. Based on the results of the mixed effects model, the trend in perceived improvement in accessibility of harm reduction services was statistically significant (aOR 0.66; *p* = 0.000, 95% CI 0.57–0.78)—see Table 2.

Qualitative interviews indicated that access to needle and syringe programs became problematic because harm reduction sites reduced working hours and had to comply with social distancing requirements. Provision of voluntary counselling and testing services was particularly affected. Those respondents who were using harm reduction services noted that programs made reasonable adjustments to their operations and employed flexible strategies to ensure continuous provision of services. For example, services extensively used mobile vans and prioritized offering HIV self-testing to their clients. One respondent noted that he would call his social worker who would bring sterile equipment to his place:

*Access was limited at the beginning. They (needle/syringe programs) worked for two days a week only and you needed to go at that time to get your syringes…*

*Yes, when offices were closed, I was calling my social worker, so he would bring syringes to my home.*

Finally, key informants shared their observation that the overall demand for OAT increased in April and May 2020. Among other factors, the rise in demand was related to the lack of traditional opioids on the market, but also to the attractiveness of OAT with at-home dosing.

As a summary of the key findings of the study, Table 3 provides presentation of the key results of our mixed methods approach.

**Table 3:** A joint presentation of a mixed methods results—key statistically significant quantitative findings and relevant illustrative/explanatory qualitative results

Quantitative findings (statistically significant trends)	Explanatory/illustrative qualitative results
Reduction in use of cannabis, alcohol, amphetamine, medicinal methadone, medicinal buprenorphine, Ketamine, LSD, myorelaxants	Difficulties in obtaining preferred substances, lack of mediating environment (night clubs, festivals), overall “depressive” context of the pandemic
*Fewer different substances used*	
Perceived access to the main drug became harder at the initial stage of the lockdown and returned to “normal” towards the end of the monitoring period	Affected contacts with dealers, lack of transportation and restrictions for movements (curfew)
Perceived increase in prices at the beginning of the lockdown and stabilization of prices (or return to initial figures) following the removal of restrictions	Distortion of conventional supply chains, middleman “fee”, heavy adulteration
Injection-related risky practices increased during the initial phase of the lockdown. As access to sterile apparatus improved, PWID returned to safer injection behaviours	Lack of access to sterile injection equipment, new practice of supply in preloaded syringe
Access to harm reduction services was strongly affected during the initial phase of the lockdown and improved later as providers adopted flexible approaches and models of service provision	Closure of provider organizations followed by the adoption of innovative service delivery models

## Discussion

Results of our study suggests that COVID-19 related restrictive measures and changes in illicit market dynamics resulted in shifts in drug use behaviours. Many PWUD switched to alternative substances, when their preferred drugs were not readily available. In some cases, these were substances that were tried/used at some previous point in a drug career, in other cases PWUD would use completely new (for them) substances. Some PWUD used drugs more frequently *“due to plenty of free time and boredom”*, but also to cope with stress, anxiety and isolation. Others reduced the frequency of drug use due to the difficulties in obtaining their favourite drugs, or because the setting in which their drug use usually occurred (night clubs and music festivals) was not there anymore, due to mandated restrictions on gatherings in such settings, as part of COVID-19 control efforts. These shifts in drug using behaviours among nightlife attendees have been noted by other authors; Zaami et al. suggest that during the period of confinement, individuals who used drugs in a nightlife settings may reduce their use, but might be looking for other drugs to be used in a private setting [43]. European data indicate that use of MDMA and cocaine (common party drugs) may have declined due to the closure of nightclubs, while use of cannabis has remained relatively stable [2, 9]. The US-based studies suggest that although recreational drug use may have declined due to COVID-19-related restrictions, many electronic dance music partygoers who use drugs would attend virtual raves and virtual happy hours [8]. Use of alcohol was highly prevalent and use of cannabis was reported by 30% of participants attending these events [8]. A cross-European study that analysed wastewater samples in seven cities in the Netherlands, Belgium, Spain and Italy at the beginning of lockdowns (March–May 2020) found that there was a decline (if compared to previous years) in illicit drug consumption for some substances and locations (e.g., 50% decrease in MDMA use), but in other cases the drug use remained stable or even increased [44]. In the studies in Germany and Switzerland, most participants reported no change in their drug consumption, and authors concluded that (at least at the early stage of the pandemic) enforced restrictions did not substantially impact the demand and consumption of illicit drugs [45, 46]. Overall, the situation in the early COVID-19 period was highly heterogenous in most of the jurisdictions for which data were available, so it was difficult to draw definite conclusions regarding the impact of the pandemic on illicit drug use.

In our study, there was a general perception among PWUD respondents and key informants that it was more difficult to obtain drugs, especially during the strictest lockdown, compared to the pre-COVID-19 time. The main reasons cited were the lack of transportation, increased police presence, and difficulties to maintain contacts with drug dealers. However, opinions with regard to which drugs availability was affected most were mixed. Overall, our data suggest that the perception of a reduced availability did not correlate much with the actual use of particular substances. This in part might be explained by the fact that in many cases individuals were able to find alternatives to their preferred substances when they became unavailable. Our findings regarding perceived changes in the price and quality of drugs were not definitive, although the majority of participants believed that the overall tendency was an increase in prices and decrease in a quality of substances available on the market. However, the quantitative data reported by participants on prices paid for specific drugs did not show any significant trends. Interpretation of these results needs to be made with caution, since an “average single dose” (as it was formulated in our questionnaire) for most substances can vary. In addition, at certain assessment points, some substances (e.g., inhalants, synthetic cannabinoids, and opium) were used by a very few respondents, which makes interpretation more challenging. Preliminary data from the E.U. suggest that supply shortages during the pandemic resulted in temporary increases in the cost of cannabis, cocaine, and some other products on the illicit drug market in Europe [9]. In an Australian study, market indicators (use of specific drugs, perceived availability and purity) remained relatively stable for most drugs, although there was some evidence of perceived reduction in the availability and purity of cocaine, methamphetamine and MDMA [47]. Authors suggested that reduction in the use of MDMA and other stimulants was mainly associated with the impediments to socialization. Similar to these findings, the study in Switzerland did not find any significant changes in the purity and availability of drugs on a local drug market [46].

Study participants noted an increase in the availability of diverted medicinal methadone and buprenorphine on the market, which followed from an unprecedented—for Georgia—decision to allow for 5-day take-home dosing of these medications for all OAT patients. Both substances were diverted by OAT patients, and were distributed free to friends or sold through personal networks of people who use drugs. The scale of this phenomenon however remains unclear. It is also unclear whether this development initiated drug use by new users, or relapse among those with a history of drug use. Results of this study did not show any increase in the use of diverted substitution medications in the study sample. Both medicinal methadone and buprenorphine were used by the study cohort (i.e., were available on the market) prior to the change in take-home dosing regulations, and the rates did not increase over the course of the study. On the contrary, the prevalence of use of medicinal methadone declined by the time of the last interview session. Again, it is difficult to unambiguously determine if this decline was caused by the reduction in the availability of medicinal methadone on the market after take-home dosing was banned again in the beginning of September 2020. In a Canadian study, authors suggest that following the adoption of flexible protocols and expansion of take-home dosing, the reduction in directly observed dosing may have posed a risk to public safety, due to diversion of prescribed medication and its non-medical consumption [3]. In a US-based study, authors reported similar concerns from the side of OAT providers [48]. Respondents (OAT care providers) in that study also expressed concerns over the increased risk for overdose given that some clients might have consumed higher doses of OAT medication or use illicit drugs in combination. However, the study in Australia did not find an association between the expansion of take-home dosing and increase in substance use [49]. Obviously, there is a need to find a balance between strict infection control measures and monitoring, to mitigate the risk for negative health effects and diversion.

We identified certain changes in drug supply models. For example, for drugs that were purchased through online platforms via dead drops, drop locations were moved to Tbilisi (capital city) suburbs and isolated locations to avoid detection by the law enforcement while police presence was intensified in central districts of the city. When stable contacts with dealers were adversely affected by pandemic-related restrictions, users searched for new contacts and supply options. In doing so, previously closed PWUD networks started interacting with each other, and in some cases merged, in an attempt to identify new channels of drug supply. PWUD with financial resources recruited middlemen, in order to reduce legal risks associated with illicit transactions and to limit their exposure to virus transmission. Overall, the role of middlemen increased, and they seemingly became important players in the altered drug market landscape. Again, this study highlighted the role of a social supply in the Georgian drug scene—a significant share of reported drug interactions occurred as a free giving/sharing among friends and members of a social network. Similar to our findings, researchers in Germany reported a specific change in drug distribution methods. While before COVID-19 customers were going to their dealers to procure drugs, following the introduction of restrictions, drug dealers adapted supply methods and began deliver drugs (drop off) to their clients [50]. Our findings, along with the findings of other authors indicate that drug market players (both dealers and consumers) showed remarkable flexibility while adjusting to altered drug market landscape and market conditions.

Results of this study suggest that when access to sterile injection equipment was limited due to restrictions on movement and scaled down provision of harm reduction services, PWUD exercised risk-containing injection behaviours. The first weeks of lockdown were accompanied by a rise in risky practices, in particular receiving used syringe and sharing instruments and tools for drug preparation and division. Such practices, however, were abandoned as soon as lockdown measures were relaxed and access to sterile equipment restored. In other words, individuals who consume drugs through injection showed awareness of risk limiting practices and demonstrated their ability to take care of their health when basic supportive services were available. These results are consistent with the data of the Georgian Harm Reduction Network (GHRN), which is the major provider of low-threshold harm reduction services to PWUD in the country. GHRN data show a reduction in HIV testing rates and in distribution of sterile injection equipment in March–May 2020. Countrywide, there was a six-fold reduction in a number of HIV rapid tests performed in April compared to February 2020 (360 vs. 2,221). The number of sterile syringes distributed by harm reduction sites declined by 30% in May compared to March (244,011 vs. 332,419) [51]. These findings underscore the critical importance of uninterrupted services provision to vulnerable populations, and the challenges that service providers face with providing continuous care. Available research indicates that in many countries, PWUD experienced reduced access to their usual drug-related services during the pandemic, including limited access to OAT and testing for blood-borne infections [1]. In the UK, following the implementation of stay-at-home restrictions, the number of visits to the needle and syringe programs (NSP), as well as the number of needles distributed, dropped by 30% [12]. In Spain, harm reduction centres had to reduce operating hours and work at reduced capacity during the lockdown; testing for infectious diseases and access to care were seriously disrupted [13]. In the US, of the 65 NSPs queried, 10 discontinued all operations, and 16 switched to mobile operations [14]. Most programs that remained open operated under restricted hours and the majority stopped testing for HIV and HCV. In another US-based study, authors report that approximately three-quarters of NSPs decreased or stopped providing on-site HIV and HCV testing [52].

Our findings indicate that despite some interruptions in service delivery during the first weeks of lockdown, harm reduction programs showed remarkable flexibility and were able to effectively deliver services. The useful approaches included using mobile vans more extensively, increased use of self-testing and delivering prevention equipment to the clients’ places. The use of syringe vending machines (SVMs) in Tbilisi sharply increased at this time—the number of sterile kits distributed through SVMs increased by 80% in April compared to February [53, 54]. In this situation, SVMs proved to be an effective means for uninterrupted provision of sterile equipment while ensuring no-contact service delivery. Detoxification treatment was also affected during the strictest lockdown measures. Use of these services, however, recovered when those measures were lifted. Demand for OAT increased when pandemic-related restrictions were enforced. Apparently, reduced access to illicit drugs and attractiveness of take-home dosing were drivers that contributed to the rise in demand. OAT programs were able to adjust quickly and effectively to changed circumstances; new clients were admitted to treatment and take-home dosing was implemented. Similar to our findings, international research indicates that the utilization of novel forms of service delivery was broadly adopted during the pandemic. For example, the use of telemedicine increased dramatically over the first months of implementation of COVID-19-related restrictive measures [55, 56]. Innovative solutions have been introduced to reduce viral transmission risk by reducing physical contact between providers and clients, for example MySafe devices for dispensing medications through a confirmatory biometric scan of the palm [57], virtual individual or group therapy sessions [58], home delivery of services (e.g., expansion of home-based and mail-based naloxone delivery) [52], and expanded uptake of long-acting medications [58].

### Limitations

Our quantitative data were collected via online platform, and we had no means to verify the identity of participants in the bi-weekly surveys. However, we did not identify major inconsistencies across the longitudinal data points and believe that the risk of someone else (not the study participant) filling out the questionnaire was minimal to non-existent. Our data are limited to the first six months of COVID-19 lockdown, and thus, the observed trends might have changed after the data collection period. The small sample (*n* = 50) reduced our ability to detect the effect. In addition, the study sample, consisting of the residents of the capital city with easy access to mobile technology, may not be representative of all people who use drugs in Georgia. Other individuals who live in remote areas, might have responded differently to the changes in the context overall, and in illicit drug markets in particular. In addition to the small sample size, some drugs were used infrequently, which might further affect generalizability of the results, specifically with regard to the less popular used drugs. Nevertheless, our aim was not to obtain statistically robust estimates of prevalence of specific types of drug use. We instead aimed to detect changes in behaviours of PWUD and to understand the factors that drive those changes. The mixed method approach used for this study allowed us to achieve synergy and provide better understanding of the observed trends and the rationale behind them. The qualitative data collected from the field experts (key informants) contributed to this approach and provided valuable insights.

## Conclusion

This study contributes to the understanding of how drug use behaviours and service provision adapted to the extraordinary situation caused by the COVID-19 pandemic. Changes in supply models and drug-use behaviours indicate that many PWUD will engage in activities that put them under the increased risks of overdose and of acquiring blood-borne infection.

There are several important public health implications that can be drawn based on this study. Harm reduction and treatment services can improve sustainability of service delivery by implementing clear protocols that can be enforced in any future response to epidemics or other emergency situations. Such protocols should build upon the experience accumulated during the COVID-19-related restrictions, such as flexible dosing of medication, utilization of HIV self-testing technologies, mobile van-based outreach, and vending machines for dispensing sterile injection equipment. In addition, prevention and education components of these services should include information to raise the awareness of PWUD about the risks identified in this study, such as increased injections from pre-filled syringes. OAT programs need to develop and implement clear and flexible procedures for medication take-home dosing, to ensure a balanced approach to medication dispensing practices while controlling risks for diversion and poor patient outcomes.

## Supplementary Information

**Additional file 1**. Study questionnaire and outcome variables, measures, and assessment time points.
