# Supplementary material for: The impact of the COVID-19 pandemic on illicit drug supply, drug-related behaviour of people who use drugs and provision of drug related services in Georgia: results of a mixed methods prospective cohort study
Source: Harm Reduct J. 2022 Mar 9;19:25. doi: 10.1186/s12954-022-00601-z (PMC8906357; doi:10.1186/s12954-022-00601-z)
Supplement: Supplementary file 1 — Additional file 1. Study questionnaire and outcome variables, measures, and assessment time points. [file 12954_2022_601_MOESM1_ESM.pdf]

Table 1. Outcome variables, measures and assessment time points.

Outcome variable	Measure	Time point
Socio-demographic characteristics	Age	Week 0 (baseline)
	Gender	Week 0
	Education	Week 0
	Employment status	Week 0
	Source of income	Weeks 0-24 (0, 2, 4, 6, 8, 10, 12, 14, 16, 18, 20, 22, 24)
Drug use	Drugs ever used	Week 0
	Age of first use (by drug)	Week 0
	Dugs used in the past 12 months	Week 0
	Dugs used in the past 14 days	Weeks 0-24
	Route of administration (by drug)	Weeks 0-24
Drug supply	Source of drugs (by drug)	Weeks 0-24
	Price paid/dose	Weeks 0-24
	Perceived change in price (increase/no change/decrease)	Weeks 0-24
	Perceived change in quality (increase/no change/decrease)	Weeks 0-24
	Perceived access to drugs (easier/no change/more difficult)	Weeks 0-24
Frequency of use	Times/day	Weeks 0-24
	Days/week	Weeks 0-24
History of overdose	Number of overdoses ever	Week 0
	Number of overdoses in the past 14 days	Weeks 0-24
	Drug caused last overdose	Weeks 0-24
Drug treatment experience	Number of treatment episodes ever (by type)	Week 0
	Currently in treatment (by type)	Weeks 0-24
	Treatment initiation in the past 14 days (by type)	Weeks 0-24
	Number of days in treatment in the past 14 days (by type)	Weeks 0-24
Risk behavior related to the spread of infections	Frequency shared needle/syringe in the past 14 days	Weeks 0-24
	Receiving (yes/no) already filled in syringe in the past 14 days	Weeks 0-24
	Using syringe (yes/no) filled from other syringe in the past 14 days	Weeks 0-24
	Sharing injection instruments (yes/no) in the past 14 days	Weeks 0-24
	Frequency having clean needle/syringe for each injection in the past 14 days	Weeks 0-24
	Frequency shared vaporizer, inhaler, tube, pipe in the past 14 days	Weeks 0-24
Access to harm reduction	Source of clean needles/syringes in the past 14 days	Weeks 0-24
	Reasons not having clean needles/syringes in the past 14 days	Weeks 0-24
	Perceived change in access to harm reduction (easier/no change/more difficult)	Weeks 0-24
COVID-19 experience	Being tested for COVID (yes/no) and test results (-/+)	Weeks 0-24
	Being hospitalized for COVID (yes/no) and number of days	Weeks 0-24

Study questionnaire

Q1. Age:

Select the correct answer from the listing below and write the answer number in the box

Q2. Gender:

1. Male
2. Female
3. Nonbinary

Q3. What is the highest level of education you completed:

1. No education
2. Completed primary school (grades 1-4)
3. Incomplete high school education (grades 5-9)
4. Completed high school education (grades 10-12)
5. Completed high school-vocational education (technical college)
6. Incomplete university education (completed third year)
7. Completed university

Q4. Employment:

1. Unemployed
2. Employed (full time, part time, self-employed)
3. Student and employed
4. Student
5. Retired/social benefits

**Please insert X in the relevant cell to indicate "yes" or "no" for every substance listed; indicate the age of first use in the last column**	**Q5. Have you ever used without doctor's prescription**		**Q6. Age of the first use**
	yes	no	
1. Heroin, syrets			
2. Opium, black, shirka			
3. Street Methadone			
4.Methadone from program			
5. Street Subutex/Suboxone			
6. Subutex/Suboxone from program			
7. Cocaine, crack			
8. Vint, jeff, Ephedr/Connifera vint			
9. Amphetamine, metamphetanine(pill, powder, cristal)			
10. Cannabis (marijuana, hashish)			
11. Alcohol			
12. Ecstasy, MDMA			
13. NBOMe			
14. Ketamine			
15. LSD			
16. Other hallucinogens (mushrooms, psilocybin, belladonna)			
17. Volatile solvents, inhalants (glue, benzene, "rastvaritel")			
18. Psychotropic myorelaxants (Lirica, Gabba-gamma, Baclosan, Gabapentine, Rivotril)			
19. Psychotropic, sedatives, tranquilizers (benzos, Diazepam, Relanium, Fenasepam, Ciclodol)			
20. Antihistamines, alone or as additions to the main drug (Dimedrol, Pipolphen, Suprastin)			
21. Synthetic cannabinoids (spices)			
22. Synthetic cathinones (bath salts, PVP, alpha-PVP, MDPV, Mephedrone)			
23. Other			

**Q7. Indicate which substance you used in the past 12 months without doctor's prescription prior to March 2020, and indicate what was the usual route of administration for each substance** [display only substances checked in Q5]							
	1. injection	2. Drinking/Swallowing/eating	3. smoking	4. vaporizing	5. snorting	6. inhaling	7. supra/sub-lingual
1. Heroin, syrets							
2. Opium, black, shirka							
3. Street Methadone							
4.Methadone from program							
5. Street Subutex/Suboxone							
6. Subutex/Suboxone from program							
7. Cocaine, crack							
8. Vint, jeff, Ephedr/Connifera vint							
9. Amphetamine, metamphetanine(pill, powder, cristal)							
10. Cannabis (marijuana, hashish)							
11. Alcohol							
12. Ecstasy, MDMA							
13. NBOMe							
14. Ketamine							
15. LSD							
16. Other hallucinogens (mushrooms, psilocybin, belladonna)							
17. Volatile solvents, inhalants (glue, benzene, "rastvaritel")							
18. Psychotropic myorelaxants (Lirica, Gabba-gamma, Baclosan, Gabapentine, Rivotril)							
19. Psychotropic, sedatives, tranquilizers (benzos, Diazepam, Relanium, Fenasepam, Ciclodol)							
20. Antihistamines, alone or as additions to the main drug (Dimedrol, Pipolphen, Suprastin)							
21. Synthetic cannabinoids (spices)							
22. Synthetic cathinones (bath salts, PVP, alpha-PVP, MDPV, Mephedrone)							
23. Other							

	**Q8. Indicate where from you usualy obtained the substance prior to March 2020 (check up to 2 sources, however indicating ONE main source would be preferable)** [display only substances checked in Q7]							
	1. Buying through internet and	2. Buying via digital app and receiving coordinates of place where the	3. Buying from dealer face-to-face	4. Buying from intermediary/friend/acquaintance	5. Receiving for free from friend/acquaintance	6. Producing/cooking/growing myself	7. Psychotropic pharmacy	8. Other

	receiving by post	drug was hidden in advance						
1. Heroin, syrets								
2. Opium, black, shirka								
3. Street Methadone								
4.Methadone from program								
5. Street Subutex/Suboxone								
6. Subutex/Suboxone from program								
7. Cocaine, crack								
8. Vint, jeff, Ephedr/Connifera vint								
9. Amphetamine, metamphetanine(pill, powder, cristal)								
10. Cannabis (marijuana, hashish)								
11. Alcohol								
12. Ecstasy, MDMA								
13. NBOMe								
14. Ketamine								
15. LSD								
16. Other hallucinogens (mushrooms, psilocybin, belladonna)								
17. Volatile solvents, inhalants (glue, benzene, “rastvaritel”)								
18. Psychotropic myorelaxants (Lirica, Gabba-gamma, Baclosan, Gabapentine, Rivotril)								
19. Psychotropic, sedatives, tranquilizers (benzos, Diazepam, Relanium, Fenasepam, Ciclodol)								
20. Antihistamines, alone or as additions to the main drug (Dimedrol, Pipolphen, Suprastin)								
21. Synthetic cannabinoids (spices)								
22. Synthetic cathinones (bath salts, PVP, alpha-PVP, MDPV, Mephedrone)								
23. Other								

	**Q9. (If buying a drug or ingredients for preparing a drug) How much did you pay for your single average dose prior to March 2020 (in national currency)** [display only substances checked in Q7]
1. Heroin, syrets	
2. Opium, black, shirka	

3. Street Methadone	
4.Methadone from program	
5. Street Subutex/Suboxone	
6. Subutex/Suboxone from program	
7. Cocaine, crack	
8. Vint, jeff, Ephedr/Connifera vint	
9. Amphetamine, metamphetanine(pill, powder, cristal)	
10. Cannabis (marijuana, hashish)	
11. Alcohol	
12. Ecstasy, MDMA	
13. NBOMe	
14. Ketamine	
15. LSD	
16. Other hallucinogens (mushrooms, psilocybin, belladonna)	
17. Volatile solvents, inhalants (glue, benzene, “rastvaritel”)	
18. Psychotropic myorelaxants (Lirica, Gabba-gamma, Baclosan, Gabapentine, Rivotril)	
19. Psychotropic, sedatives, tranquilizers (benzos, Diazepam, Relanium, Fenasepam, Ciclodol)	
20. Antihistamines, alone or as additions to the main drug (Dimedrol, Pipolphen, Suprastin)	
21. Synthetic cannabinoids (spices)	
22. Synthetic cathinones (bath salts, PVP, alpha-PVP, MDPV, Mephedrone)	
23. Other	

**Report here ONLY on substances that you used regularly (at least once a week) for 3 months prior to March 2020** [display only substances checked in Q7]		
	**Q 10. Prior to March 2020, as usual, how many times a day did you use**	**Q 11. Prior to March 2020, as usual, how many days a week did you use**
1. Heroin, syrets		
2. Opium, black, shirka		
3. Street Methadone		
4.Methadone from program		
5. Street Subutex/Suboxone		
6. Subutex/Suboxone from program		
7. Cocaine, crack		
8. Vint, jeff, Ephedr/Connifera vint		
9. Amphetamine, metamphetanine(pill, powder, cristal)		
10. Cannabis (marijuana, hashish)		
11. Alcohol		
12. Ecstasy, MDMA		
13. NBOMe		
14. Ketamine		
15. LSD		
16. Other hallucinogens (mushrooms, psilocybin, belladonna)		
17. Volatile solvents, inhalants (glue, benzene, “rastvaritel”)		
18. Psychotropic myorelaxants (Lirica, Gabba-gamma, Baclosan, Gabapentine, Rivotril)		
19. Psychotropic, sedatives, tranquilizers (benzos, Diazepam, Relanium, Fenasepam, Ciclodol)		
20. Antihistamines, alone or as additions to the main drug (Dimedrol, Pipolphen, Suprastin)		
21. Synthetic cannabinoids (spices)		
22. Synthetic cathinones (bath salts, PVP, alpha-PVP, MDPV, Mephedrone)		
23. Other		

Q12. How many times had you overdosed on drugs in your lifetime? [skip to Q14 if “0”]

Q13. What substance was the cause of overdose at the last overdose episode? [display only substances checked in Q5]

Q14. How many times have you ever received treatment due to your drug problems?

		Number of treatment episodes	Check if currently in treatment
0	No treatment		
1	Outpatient detoxification		
2	Detoxification residential		
3	Outpatient substitution		
4	Outpatient drug-free		
5	Drug-free residential		
6	Day care		
7	Psychiatric hospital		
8	Other hospital/ward		
9	Other treatment		

Q15. How would you describe your main source of income BEFORE March 2020 [choose all that apply]?

1 Full time / part time job
2 Seasonal / temporary job
3 Social payments (stipend, pension)
4 Support of other people (spouse, other relatives)
5 Illegal activities
6 Other

Q16. Indicate which substance did you use during the past 14 days without doctor's prescription and by what rout

	1. injection	2. Drinking/Swallowing/eating	3. smoking	4. vaporizing	5. snorting	6. inhaling	7. supra/sub-lingual
1. Heroin, syrets							
2. Opium, black, shirka							
3. Street Methadone							
4.Methadone from program							
5. Street Subutex/Suboxone							
6. Subutex/Suboxone from program							
7. Cocaine, crack							
8. Vint, jeff, Ephedr/Connifera vint							
9. Amphetamine, metamphetanine(pill, powder, cristal)							
10. Cannabis (marijuana, hashish)							
11. Alcohol							
12. Ecstasy, MDMA							
13. NBOMe							
14. Ketamine							
15. LSD							
16. Other hallucinogens (mushrooms, psilocybin, belladonna)							
17. Volatile solvents, inhalants (glue, benzene, "rastvaritel")							
18. Psychotropic myorelaxants (Lirica, Gabba-gamma, Baclosan, Gabapentine, Rivotril)							
19. Psychotropic, sedatives, tranquilizers (benzos, Diazepam, Relanium, Fenasepam, Ciclodol)							
20. Antihistamines, alone or as additions to the main drug (Dimedrol, Pipolphen, Suprastin)							
21. Synthetic cannabinoids (spices)							

22. Synthetic cathinones (bath salts, PVP, alpha-PVP, MDPV, Mephedrone)							
23. Other							

	**Q17. Indicate where from you usualy obtained the substance during the past 14 days (check up to 2 sources, however indicating ONE main source would be preferable)** [display only substances checked in Q16]							
		1. Buying through internet and receiving by post	2. Buying via digital app and receiving coordinates of place where the drug was hidden in advance	3. Buying from dealer face-to-face	4. Buying from intermediary/friend/acquaintance	5. Receiving for free from friend/acquaintance	6. Producing/cooking/growing myself	7. Psychotropic pharmacy	8. Other
1. Heroin, syrets								
2. Opium, black, shirka								
3. Street Methadone								
4.Methadone from program								
5. Street Subutex/Suboxone								
6. Subutex/Suboxone from program								
7. Cocaine, crack								
8. Vint, jeff, Ephedr/Connifera vint								
9. Amphetamine, metamphetanine(pill, powder, cristal)								
10. Cannabis (marijuana, hashish)								
11. Alcohol								
12. Ecstasy, MDMA								
13. NBOMe								
14. Ketamine								
15. LSD								
16. Other hallucinogens (mushrooms, psilocybin, belladonna)								
17. Volatile solvents, inhalants (glue, benzene, “rastvaritel”)								
18. Psychotropic myorelaxants (Lirica, Gabba-gamma, Baclosan, Gabapentine, Rivotril)								
19. Psychotropic, sedatives, tranquilizers (benzos, Diazepam, Relanium, Fenasepam, Ciclodol)								
20. Antihistamines, alone or as additions to the main drug (Dimedrol, Pipolphen, Suprastin)								
21. Synthetic cannabinoids (spices)								

22. Synthetic cathinones (bath salts, PVP, alpha-PVP, MDPV, Mephedrone)									
23. Other									

	**Q 18. (If buying a drug or ingredients for preparing a drug) for the past 14 days how much did you pay for your single average dose (in national currency)**
1. Heroin, syrets	
2. Opium, black, shirka	
3. Street Methadone	
4.Methadone from program	
5. Street Subutex/Suboxone	
6. Subutex/Suboxone from program	
7. Cocaine, crack	
8. Vint, jeff, Ephedr/Connifera vint	
9. Amphetamine, metamphetanine(pill, powder, cristal)	
10. Cannabis (marijuana, hashish)	
11. Alcohol	
12. Ecstasy, MDMA	
13. NBOMe	
14. Ketamine	
15. LSD	
16. Other hallucinogens (mushrooms, psilocybin, belladonna)	
17. Volatile solvents, inhalants (glue, benzene, “rastvaritel”)	
18. Psychotropic myorelaxants (Lirica, Gabba-gamma, Baclosan, Gabapentine, Rivotril)	
19. Psychotropic, sedatives, tranquilizers (benzos, Diazepam, Relanium, Fenasepam, Ciclodol)	
20. Antihistamines, alone or as additions to the main drug (Dimedrol, Pipolphen, Suprastin)	
21. Synthetic cannabinoids (spices)	
22. Synthetic cathinones (bath salts, PVP, alpha-PVP, MDPV, Mephedrone)	
23. Other	

	**Q19. During the past 14 days, as usual, how many times a day did you use**	**Q20. During the past 14 days, as usual, how many days a week did you use**
1. Heroin, syrets		
2. Opium, black, shirka		
3. Street Methadone		
4.Methadone from program		
5. Street Subutex/Suboxone		
6. Subutex/Suboxone from program		
7. Cocaine, crack		
8. Vint, jeff, Ephedr/Connifera vint		
9. Amphetamine, metamphetanine(pill, powder, cristal)		
10. Cannabis (marijuana, hashish)		
11. Alcohol		
12. Ecstasy, MDMA		
13. NBOMe		
14. Ketamine		
15. LSD		
16. Other hallucinogens (mushrooms, psilocybin, belladonna)		
17. Volatile solvents, inhalants (glue, benzene, “rastvaritel”)		

18. Psychotropic myorelaxants (Lirica, Gabba-gamma, Baclosan, Gabapentine, Rivotril)		
19. Psychotropic, sedatives, tranquilizers (benzos, Diazepam, Relanium, Fenasepam, Ciclodol)		
20. Antihistamines, alone or as additions to the main drug (Dimedrol, Pipolphen, Suprastin)		
21. Synthetic cannabinoids (spices)		
22. Synthetic cathinones (bath salts, PVP, alpha-PVP, MDPV, Mephedrone)		
23. Other		

Q21. How many times have you overdosed on drugs during the past 14 days? [skip to Q23 if "0"]

Q22. What substance was the cause of overdose at the last overdose episode? [display only substances checked in Q16]

Q23. Have you initiated or continued drug treatment during the past 14 days? [skip to Q25 if "0"]

		Continued (yes/no)	Initiated (yes/no)
0	No treatment		
1	Outpatient detoxification		
2	Detoxification residential		
3	Outpatient substitution		
4	Outpatient drug-free		
5	Drug-free residential		
6	Day care		
7	Psychiatric hospital		
8	Other hospital/ward		
9	Other treatment		

Q24. How many days of the past 14 days have you received treatment? [display treatments checked in Q23]

		Number days in treatment
1	Outpatient detoxification	
2	Detoxification residential	
3	Outpatient substitution	
4	Outpatient drug-free	
5	Drug-free residential	
6	Day care	
7	Psychiatric hospital	
8	Other hospital/ward	
9	Other treatment	

Q25. In your opinion, how did the **price** of your main drug change in the past 14 days?

1. Became cheaper
2. Did not change
3. Became more expensive

Q26. In your opinion, how did the **quality** of your main drug change in the past 14 days?

1. Became better
2. Did not change
3. Became worse

Q27. In your opinion, how did the **access to** your main drug change in the past 14 days?

1. Became easier to get
2. Did not change
3. Became harder to get

Q28. In your opinion, how did **access to harm reduction programs** change in the past 14 days?

1. Became easier

2. Did not change
3. Became worse
4. Did not use harm reduction programs

Q29. Think about the times you injected drugs in the past 14 days. How often was it with a needle or syringe that had been previously used by someone else?

1. Always
2. Most times
3. About half the time
4. Occasionally
5. Never
6. I did not inject in the past 14 days [skip to Q36]

Q30. Have you received/ bought an injection from an already filled syringe (i.e. you did not see how it was filled) in the last 14 days?

1. Yes
0. No

Q31. In the last 14 days when you injected drugs, did you use a syringe, filled by someone from his / her already used syringe (direct or reverse filling, several doses in one syringe)?

1. Yes
0. No

Q32. Have you used common instruments for sharing (preparation) of a drug at least once in the last 14 days?

1. Yes
0. No

Q33. In the past 14 days, you had a clean needle for each injection:

1. Always
2. Most times
3. About half the time
4. Occasionally
5. Never

Q34. Where did you get clean needles/syringes in the past 14 days? (check all that apply)

1. Bought in a pharmacy
2. Received for free in a pharmacy
3. Received for free from vending machine
4. Received from a harm reduction program social worker (i.e. at a syringe exchange site, at an NGO, through outreach)
5. Got it from a friend or partner
6. Got a used syringe/needle from a friend or partner
7. Used my previously used needles/syringes
8. Bought the drug in pre-filled syringe
9. Found a used syringe/needle on the street
10. Other

Q35. What were the reasons you did not have enough clean needles/syringes for each injection in the past 14 days? (check all that apply)

0. I had clean needles/syringes for each injection
1. The pharmacy did not work as usual
2. The harm reduction program did not work as usual
3. I don't have enough money
4. The transportation did not work
5. I stayed at home because of the quarantine
6. I was sick
7. Other

Q36. Think about the times you smoked, vaporized or inhaled drugs in the past 14 days. How often did you share the device or instrument (vaporizer, tube, pipe, stem tip) with someone else (you used after someone or the other person used it after you?

1. Always
2. Most times
3. About half the time
4. Occasionally
5. Never
6. I did not smoke, vaporized or inhaled drugs in the past 14 days

Q37. How would you describe your main source of income for the past 14 days [choose all that apply]?

1. Full time / part time job
2. Seasonal / temporary job
3. Social payments (stipend, pension)
4. Support of other people (spouse, other relatives)
5. Illegal activities
6. Other

Q38. Have you been tested for COVID in the past 14 days?

1 Yes and I was negative
2 Yes and I was positive
3 Yes and I am waiting for the result
4 No

Q39. Have you been hospitalized, and for how many days, in the past 14 days?

1 Yes for suspected COVID-19 (#days)
2 Yes for other (non COVID-19 related) reasons (#days)
2 No
